# Association between Exposure to Ambient Air Pollution and the Risk of Rheumatoid Arthritis in Taiwan: A Population-Based Retrospective Cohort Study

**DOI:** 10.3390/ijerph19127006

**Published:** 2022-06-08

**Authors:** Wen-Chao Ho, Li-Wei Chou, Ruey-Yun Wang, Thanh-Nhan Doan, Hwa-Lung Yu, Ting-Hsuan Chou, Kang-Yung Liu, Po-Chang Wu, Shwn-Huey Shieh

**Affiliations:** 1 Department of Public Health, College of Public Health, China Medical University, Taichung 406040, Taiwan; wcho@mail.cmu.edu.tw (W.-C.H.); rywang@mail.cmu.edu.tw (R.-Y.W.); u110050102@cmu.edu.tw (T.-N.D.); thchou@nhri.edu.tw (T.-H.C.); u103014303@cmu.edu.tw (K.-Y.L.); 2 Department of Physical Medicine and Rehabilitation, China Medical University Hospital, Taichung 404332, Taiwan; chouliwe@mail.cmu.edu.tw; 3 Department of Physical Medicine and Rehabilitation, Asia University Hospital, Asia University, Taichung 41354, Taiwan; 4 Department of Physical Therapy and Graduate Institute of Rehabilitation Science, China Medical University, Taichung 406040, Taiwan; 5 Department of Rehabilitation, Quang Nam Northern Mountainous Region General Hospital, Quang Nam 560000, Vietnam; 6 Department of Bioenvironmental Systems Engineering, National Taiwan University, Taipei 10617, Taiwan; hlyu@ntu.edu.tw; 7 Ph.D. Program in Translational Medicine, National Chung Hsing University, Taichung 40227, Taiwan; 8 School of Medicine, College of Medicine, China Medical University, Taichung 40402, Taiwan; 9 Rong Hsing Research Center For Translational Medicine, National Chung Hsing University, Taichung 40227, Taiwan; 10 Rheumatology and Immunology Center, China Medical University Hospital, Taichung 404332, Taiwan; 11 Department of Health Services Administration, College of Public Health, China Medical University, Taichung 406040, Taiwan; 12 Department of Nursing, Asia University, Taichung 41354, Taiwan

**Keywords:** rheumatoid arthritis, air pollution, PM2.5, PM10

## Abstract

Background: The association between ambient air pollution (AAP) and the risk of Rheumatoid arthritis (RA) remains debatable. We conducted a population-based cohort study to investigate the association between exposure to AAP and the risk of RA in Taiwan. Methods: We analyzed and combined the longitudinal Health Insurance Database (LHID) and the Taiwan Air Quality-Monitoring Database (TAQMD), which were in line with the residential areas. We calculated the RA incidence rates per 10,000 person-years exposed to each quartile of PM2.5 or PM10 concentrations or RH. Hazards regression was conducted to analyze the associations between exposure to each quartile of PM2.5 and PM10 concentrations and the risk of developing RA. The hazard ratios of RA were analyzed between participants exposed to annual average concentrations of PM2.5 and PM10. All the hazard ratios of RA were stratified by gender and adjusted for age and relative humidity (RH). A *p*-value < 0.05 was considered statistically significant. Results: Among 722,885 subjects, 9338 RA cases were observed. The analyses adjusted for age, gender, and humidity suggested an increased risk of developing RA in the exposure to PM2.5 in the last quartile (Q4) with the adjusted hazard ratio (aHR) was 1.053 (95%CI: 1.043 to 1.063). Conclusion: Our study suggests that exposure to PM2.5 is associated with an increased risk of RA. The finding has implications for policymaking to develop coping strategies to confront AAP as a risk factor for RA.

## 1. Introduction

Rheumatoid arthritis (RA) is one of the most common autoimmune inflammatory arthritis that causes progressive joint erosions and joint deformities; it correlates to progressive disability, numerous secondary consequences, mortality, and socioeconomic burden [1]. According to the estimates from the global burden of disease 2010 study, the worldwide prevalence of RA was reported as 0.24% [2]. More recently, a meta-analysis in 2020 showed the global prevalence of rheumatoid arthritis was 0.46% [3]. The RA prevalence estimated in European countries and the United States is typically greater, ranging from 0.5% to 1% [3,4,5]. The lowest prevalence of RA was found in Asia and South America at around 0.30% [3]. In Taiwan, the period prevalence of RA between 2002 and 2007 was 97.5 cases per 100,000 people [6], the average annual age-adjusted incidence rate was 15.8 per 100,000; additionally, there was a tendency to increase RA incidence in Taiwan [6]. Although the precise etiopathogenesis of RA has not been well elucidated, various genetic and environmental factors have been determined to be involved in a complex interplay [7]. The environmental exposures most likely occur at mucosal surfaces [8]. Chronic mucosal inflammation, including the oral and respiratory mucosa appeared to be associated with RA [9,10]. The progress of RA takes place over years, with evidence of autoimmunity developing gradually. A number of environmental factors contribute to RA susceptibility, wherein smoking has been recognized as the most important causative factor for RA [7,11,12]. Cigarette smoking induces peptidyl arginine deiminase (PAD) expression in alveolar macrophages [13]. PADs convert arginine to citrulline in the airway, creating neoantigens that can activate the adaptive immune system, which leads to the production of anti-citrullinated protein antibodies (ACPAs) that could initiate inflammation in synovium [8]. Ambient air pollution is a mixture of solid particles (particulate matter of various sizes) and gases (NO_x_, SO_2_, O_3_, or CO, etc.) in the air [14]. There has been increasing evidence that exposure to air pollution is closely associated with the incidence and development of autoimmune diseases including RA [15,16]. The putative mechanisms include systemic inflammation, increased oxidative stress, epigenetic modifications induced by air pollution exposures, and immune response to airway damage [17]. The chronic respiratory damage that occurs in an individual with a genetic background may facilitate the development of autoimmunity, leading to autoimmune diseases. A higher incidence of RA has been reported in residential proximity to traffic, after adjustment for multiple confounders, in a prospective cohort study of 90,297 U.S. women in a Nurses’ Health Study [18]. These associations between RA and living near traffic were then further confirmed in a study conducted in Canada [19]. Particulate matter potentially causes systemic inflammation by accumulating in airways and then traveling through all the circulation; its adverse effects on the cardiovascular, pulmonary, and neurological systems have been reported [20,21,22]. A recent study showed that fine particulate matter (PM2.5) induced pulmonary microvascular damage in chronic obstructive pulmonary disease [23]. Nevertheless, only a few studies evaluated the associations between the risk of RA and ambient air pollution measured by particulate matter, which reported inconsistent findings [24,25]. Data are sparse on the associations of air pollution with RA. Therefore, we conducted a population-based cohort study to examine the association between exposure to ambient air pollution and the occurrence of RA in Taiwan.

## 2. Materials and Methods

### 2.1. Data Source

We combined the longitudinal Health Insurance Database (LHID) released from the Taiwan Bureau of National Health Insurance and the Taiwan Air Quality-Monitoring Database (TAQMD) released from the Taiwan Environmental Protection Agency (EPA). The Taiwanese National Health Insurance (NHI) program is a health insurance system that covers almost the whole population in Taiwan (99%). The database was established from National Health Insurance Research Database (NHIRD) starting in 2000. This database contains personal information of beneficiaries, their demographic data, and information on medical records such as inpatient and outpatient visits, prescriptions, and other pertaining services. We used the LHID 2000, a subset database of the NHIRD that contains registration files and original claims data for reimbursement made for one million beneficiaries, who were random sampling from the 2000 Registry for Beneficiaries (ID), with a follow-up period extending to the end of 2010. The NHI released the data of National Health Insurance, which is a representative, population-based cohort. As this study used anonymized secondary data, the ethics committee of China Medical University, Taiwan waived the informed consent requirement. The TAQMD is composed of daily concentrations of particulate matter less than 2.5 µm (PM2.5) and 10 µm (PM10) in diameter as well as weather statuses such as temperature and relative humidity (RH), which were collected from 74 ambient air quality monitoring stations scattered throughout Taiwan from 2000 to 2010. We analyzed and combined both LHID and TAQMD databases, which were in line with the residential areas.

### 2.2. Study Cohort

We retrieved admission and outpatients’ visit records. A retrospective cohort was Taiwanese patients with incident RA. The diseases are classified according to the International Classification of Diseases, Ninth Revision [ICD-9-CM code 714 except for 714.3], aged > 16 years, who initiated therapy with a disease-modifying antirheumatic drug (DMARD) from 2000 to 2010. We identified patients who had two RA diagnoses within one year and received at least one DMARD (Methotrexate, Sulfasalazine, Hydroxychloroquine, Leflunomide) prescription from the first RA diagnosis. The potential patients in this cohort were identified in the admission files, and the date of the first diagnosis of RA was used as the index date. The target population, a representative sample cohort of 995,923 participants, was randomly selected, comprising 4.34% of the total eligible population in 2000. The follow-up ended on the date of diagnosis, death, or loss of follow-up from the registry or 31 December 2010.

### 2.3. Outcome and Covariates

RA is the major outcome in this study, we collected demographic variables including age, sex. According to the onset year, we stratified age into 16 to 38 years, 39 to 60 years, and >60 years. Residential areas were classified into four levels. The yearly average PM2.5 and PM10 concentration were calculated by daily data and divided into four quartiles as Q1 (<29.76 µg/m^3^), Q2 (29.76 to 34.50 µg/m^3^), Q3 (34.50 to 39.70 µg/m^3^), Q4 (>39.70 µg/m^3^), and Q1 (<48.93 µg/m^3^), Q2 (48.93 to 60.44 µg/m^3^), Q3 (60.44 to 69.98 µg/m^3^), Q4 (>69.98 µg/m^3^), respectively.

### 2.4. Statistical Analysis

We demonstrated the demographic distribution and calculated the quartile of the PM2.5 and PM10 yearly mean concentrations of all study subjects and RA patients. We assessed the incidence among study population who had at least one-year air pollution follow-up exposure before with RA diagnosis. We calculated the RA incidence rates per 10,000 person-years exposed to each quartile of PM2.5 or PM10 concentrations or RH. We executed hazards regression analysis to predict the risk of RA in people exposed to each quartile of PM2.5 and PM10 concentrations. Finally, the hazard ratios of RA were analyzed between participants exposed to annual average concentrations of PM2.5 and PM10. All the hazard ratios of RA were stratified by gender and adjusted for age and RH. A *p*-value < 0.05 was considered statistically significant. All analyses were performed using the SAS statistical package (SAS System for Windows, Version 9.4, SAS Institute Inc., Cary, NC, USA).

## 3. Results

**Figure 1:**
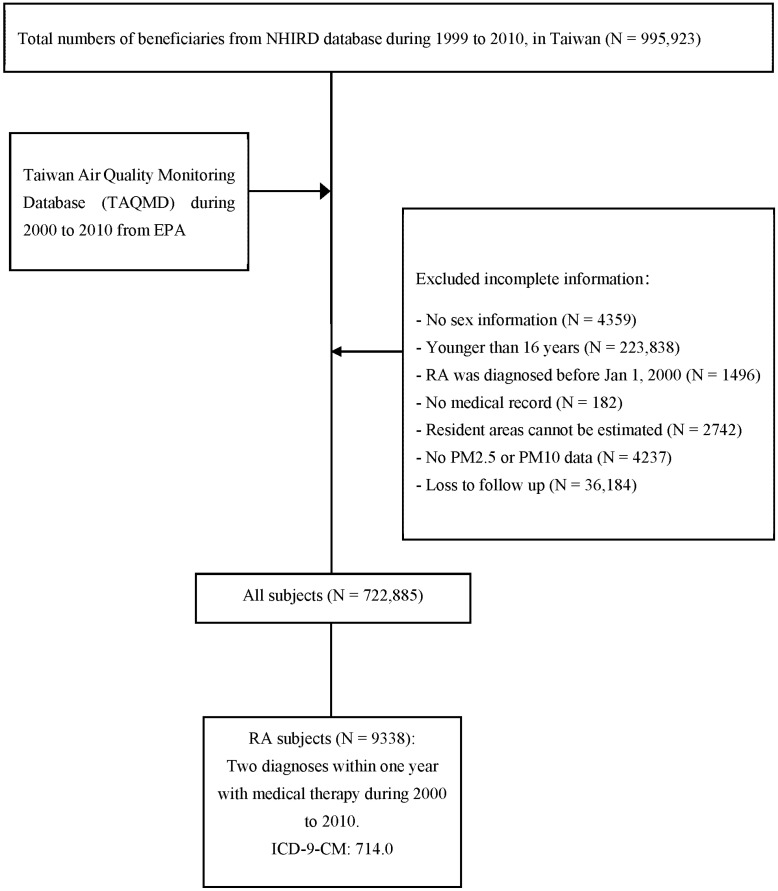
The flow chart shows the recruitment of eligible newly RA cases from 1 million beneficiaries, excluding incomplete information from 2000 to 2010, and merged Taiwan Air Quality Monitoring Database (TAQMD) files with a longitudinal cohort study.

A sample of 722,885 subjects met the eligibility criteria, in which 9338 RA patients had two RA diagnoses within one year and received at least one DMARD (Methotrexate, Sulfasalazine, Hydroxychloroquine, Leflunomide) prescription from 2000 to 2010 (Figure 1). The mean age of RA patients was 54.61 ± 15.48 years and most of them (68.56%) were females. Regarding the age distribution of RA patients, the age group 39 to 60 years accounted for the highest proportion with 48.83%, followed by the age group greater than 60 years with 36.64%, and the age group 16 to 38 accounted for the least portion with 14.53%. The distribution of RA patients in the exposure to PM2.5 was highest in Q1 (39.44%), followed by 22.61% in Q2, 20.91% in Q4, and 17.04% in Q3; the distribution of RA patients in the exposure to PM10 was highest in Q1 (41.34%), followed by 24.16% in Q3, 19.54% in Q2, and lowest in Q4 with 14.96%. Descriptive characteristics of the study population are presented in Table 1.

### 3.1. The Association between Exposure to PM2.5 and RA

With regard to the association between exposure to different PM2.5 concentrations and RA, under the control of age, gender and humidity, the highest IR was observed in Q3 (13.61). The aHR in Q4 was 1.053 (95%CI: 1.043 to 1.063), while aHR was less than 1 in Q1, Q2, and Q3. Male RA patients had the highest IR in Q3 (8.84), the aHR in Q4 was 1.052 (95%CI: 1.033 to 1.070); the highest IR in female RA patients was observed in Q3 (18.49), and the Q4 aHR was 1.053 (95%CI: 1.040 to 1.065) (Table 2).

### 3.2. The Association between Exposure to PM10 and RA

Regarding the association between exposure to different PM10 concentrations and RA, under the control of age, gender, and humidity, the highest IR was seen in Q3 (13.30). The aHR in Q4 was 1.048 (95%CI: 1.039 to 1.058), while aHR was less than 1 in Q1 to Q3. Male RA patients had the highest IR in Q3 (8.95), and the aHR of Q4 was 1.053 (95%CI: 1.036 to 1.070); female RA patients had the highest IR in Q1 of 18.20, and Q4 aHR was 1.046 (95%CI: 1.035 to 1.057) (Table 3).

## 4. Discussion

The association of smoking and the development of RA [26] have been demonstrated through epidemiologic studies [27] as well as through in vivo [28] and animal models [29] of RA. Our current study demonstrates that high exposure to PM2.5 was also associated with an increased incidence of rheumatoid arthritis (RA). RA patients had the highest IR in Q3 (13.61), and the adjusted Hazard Ratio (aHR) in Q4 was 1.053 (95%CI: 1.043 to 1.063). Female and Male RA patients had the aHR of Q4 being 1.053 (95%CI: 1.040 to 1.065) and 1.052 (95%CI: 1.033 to 1.070), respectively. The present study showed the first finding confirming that PM2.5 exposure is significantly associated with the risk of RA in Taiwan. Notably, a longitudinal study conducted in Taiwan has not shown a significant correlation between PM2.5 and the incidence of RA [30]; this previous study utilized an operational definition of RA according to the ICD-9 code. In contrast, in our study, we recruited patients who had two RA diagnoses within one year and received at least one DMARD; the discrepancy might also be produced by the controlled variables, especially relative humidity. The findings from previous studies investigating the association between PM2.5 and RA incidence across different populations were somewhat conflicting [19,24,31,32,33]. The heterogeneity might be attributable to the inconsistency in exposure measurements, operational definition, diagnosis criteria, and confounding factors. It is also known that PM2.5 differ in size, shape, surface charges, surface chemistry and chemical compositions, leading to differential health effects among particle types [34]. Our result was consistent with findings that ambient air pollution was related to autoimmune diseases [15,16,33]. The biological plausibility pertaining to the effect of air pollution on the pathogenesis of RA has been reported. Particularly, fine PM or gaseous pollutants breathed in the respiratory system would activate nuclear factor ƙappa B, leading to proinflammatory cytokine stimulation such as TNF-α and IL-6. These cytokines drive resting monocytes to develop dendritic cells, which then deliver autoantigens to self-reactive T lymphocytes, subsequently cross to target tissues (preferentially diarthrosis) and eradicate autoantigen-expressing cells [35]. Furthermore, free reactive oxygen species cause joint inflammation and erosion by stimulating chronic lung and systemic inflammation, which promotes the development of citrullinated products and anti-citrullinated protein antibodies (ACPAs) [35]. ACPAs will bind citrullinated peptides expressed on the surface of developing osteoclasts (OCs) in the nearby joints, leading to IL-8 production and bone loss. The locally produced IL-8 recruits neutrophils, followed by consecutive chemoattraction of lymphocytes to the synovial membrane and activation of synovial fibroblast to result in chronic synovitis and ACPA-positive RA [36]. A recent study in China reported that females and older adults appeared more vulnerable to PM2.5 exposure, and high-concentration PM2.5 exposure was significantly associated with an increased risk of RA rehospitalization [37]. Such a significant association between PM2.5 and RA was also found in the recent systematic review and meta-analysis [38]. Another recent study conducted in Italy also showed that exposure to high levels of air pollution including PM2.5 was correlated with increased C-reactive protein levels, as well as a higher risk of developing flares of rheumatoid arthritis [39].

There were several potential limitations in this study. First, instead of personal air monitoring data, the study has used air pollution data based on Taiwan EPA highly density air monitoring station data with well-managed QA/QC control. Second, Taiwan EPA has started to collect PM2.5 data since 2005. PM2.5 during 2000–2005 was estimated by geographic information system (GIS) with the Bayesian Maximum Entropy (BME) method. The GIS prediction with PM2.5/PM10 and PM2.5/TSP ratios has shown to provide good estimations of the PM2.5 exposure levels [40]. Third, the average measures of air pollution instead of a weighted time average may underestimate the effect of air pollution on RA. Forth, the results of genetic receptor assays were unavailable. Therefore, we could not analyze the differences in the RA with different races separately. However, the Taiwan population consists of 98% of Han Chinese ethnicity. It may help this study avoid the possible confounding effects related to race.

**Figure A1:**
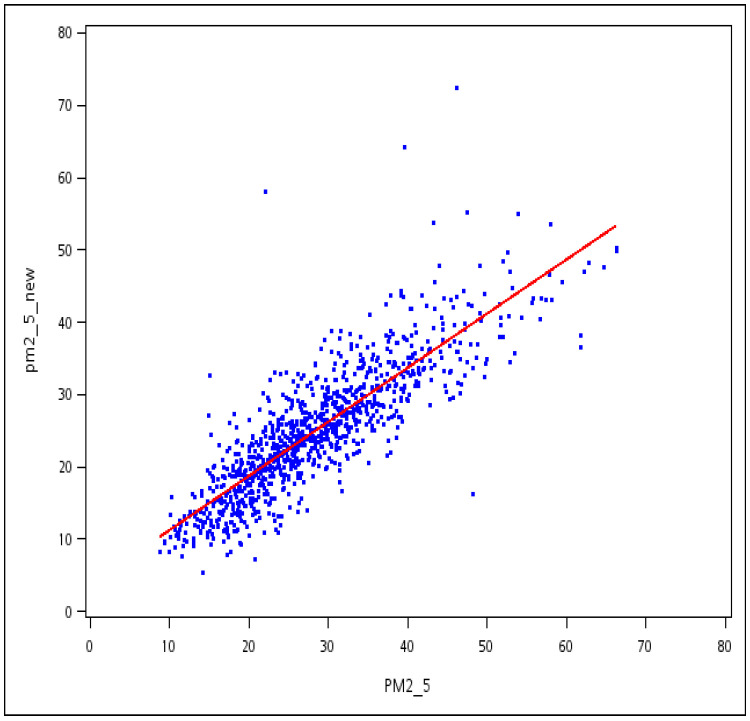
*X*-axis: land-use model, *Y*-axis: GIS (r = 0.85, n = 1003).

The strengths of this study include: First, National Health Insurance (NHI) Bureau covers most of the Taiwanese population. The potential selection bias was eliminated. Second, the RA patients have been diagnosed by clinical physicians with further double-check of NHI well-trained data administrated experts. The diagnosis was well established. Third, we gained an advantage from the high density of air-monitoring stations in Taiwan. The exposure assessment was based on and located by patients’ primary medical service activities. Fourth, the estimated PM2.5 was further verified by comparing the GIS and land-use model. Highly correlation was found (r = 0.85) (Figure A1 in Appendix A). It can provide potential well estimation of long-term PM2.5 exposure level. Furthermore, the consistent health effect of PM10 exposure has been shown and it may provide the robust comparison base.

Despite the fact that PM2.5 exposure was found to be associated with relatively small risks in this study, it could compromise a considerable number of people. The findings of this study highlighted the importance of raising awareness of the potential environmental influences and taking steps to reduce the risk of exposure. Furthermore, enhanced ambient air quality is required to minimize adverse health effects, and effective national prevention strategies against ambient air pollution need to be further firmed up and implemented.

## 5. Conclusions

In conclusion, the main finding of our study is that high exposure to PM2.5 was significantly associated with the increased incidence of rheumatoid arthritis in Taiwan. PM2.5 is a significant risk factor related to health worldwide, which compromises individuals and their families, country, and worldwide. Further study should be conducted to investigate the biological plausibility of the impact of this environmental risk factor in RA’s pathogenesis. Much remains to be done to inform policymakers better to develop coping strategies for the prevention and intervention of rheumatoid arthritis.

## Figures and Tables

**Table 1:** Baseline characteristics of participants.

Variable		ALL (N = 722,885)	%	RA (N = 9338)	%
Gender	Male	366,593	49.29	2936	31.44
	Female	356,292	50.71	6402	68.56
Age	Mean (SD)	40.34 (16.79)		54.61 (15.48)	
16–38		367,831	50.88	1357	14.53
39–60		246,643	34.12	4560	48.83
>60		108,411	15.00	3421	36.64
PM_2.5_					
Q1		276,272	38.22	3683	39.44
Q2		163,683	22.64	2111	22.61
Q3		115,099	15.92	1591	17.04
Q4		167,831	23.22	1953	20.91
PM10					
Q1		282,802	39.11	3860	41.34
Q2		148,437	20.53	1825	19.54
Q3		166,714	23.06	2256	24.16
Q4		125.32	17.30	1397	14.96
Follow years	Mean	10.27		4.7	

**Table 2:** Comparison of RA incidence and related HR differences among participants exposed to average annual concentrations of PM2.5.

Pollutant Levels	Event	PY	IR	cHR	95%CI	aHR	95%CI
All							
Q1	3683	2,844,052	12.95	0.928	0.921–0.935	0.974	0.967–0.981
Q2	2111	1,668,022	12.66	0.949	0.934–0.964	0.963	0.948–0.978
Q3	1591	1,168,749	13.61	0.979	0.966–0.993	0.946	0.934–0.968
Q4	1953	1,721,764	11.34	1.010	1.001–1.019	1.053	1.043–1.063
Male							
Q1	1101	1,391,484	7.91	0.933	0.920–0.945	0.982	0.969–0.994
Q2	681	866,044	7.86	0.962	0.937–0.987	0.976	0.953–1.000
Q3	522	590,660	8.84	0.993	0.970–1.017	0.966	0.944–0.989
Q4	632	866,440	7.29	1.019	1.002–1.035	1.052	1.033–1.070
Female							
Q1	2582	1,452,568	17.78	0.926	0.918–0.935	0.970	0.962–0.978
Q2	1430	801,978	17.83	0.941	0.922–0.961	0.955	0.936–0.973
Q3	1069	578,088	18.49	0.972	0.956–0.989	0.953	0.922–0.969
Q4	1321	855,324	15.44	1.004	0.993–1.016	1.053	1.040–1.065

PY = person-years. IR = Incidence rate, (per 10,000 person-years). cHR = crude hazard ratio. aHR = adjusted hazard ratio of multivariate analysis, after adjustment for age, sex and RH. Male and female are only adjusted for age and RH. CI = confidence interval.

**Table 3:** Comparison of RA incidence and related HR differences among participants exposed to average annual concentrations of PM10.

Pollutant Levels	Event	PY	IR	cHR	95%CI	aHR	95%CI
All							
Q1	3860	2,907,882	13.27	0.946	0.942–0.951	0.984	0.980–0.988
Q2	1825	1,513,200	12.06	0.950	0.941–0.958	0.982	0.973–0.991
Q3	2256	1,696,087	13.30	1.019	1.013–1.026	0.998	0.994–1.003
Q4	1397	1,285,417	10.87	0.998	0.991–1.005	1.048	1.039–1.058
Male							
Q1	1160	1,424,055	8.15	0.952	0.944–0.959	0.992	0.985–0.998
Q2	584	788,026	7.41	0.959	0.944–0.973	1.001	0.987–1.015
Q3	767	857,465	8.95	1.020	1.009–1.031	0.999	0.990–1.006
Q4	425	645,083	6.59	1.009	0.995–1.022	1.053	1.036–1.070
Female							
Q1	2700	1483,828	18.20	0.943	0.938–0.949	0.981	0.976–0.986
Q2	1241	725,174	17.11	0.944	0.933–0.955	0.971	0.960–0.983
Q3	1489	838,622	17.76	1.019	1.011–1.027	0.998	0.992–1.004
Q4	972	640,334	15.18	0.993	0.984–1.001	1.046	1.035–1.057

PY = person-years. IR = Incidence rate, (per 10,000 person-years). cHR = crude hazard ratio. aHR = adjusted hazard ratio of multivariate analysis, after adjustment for age, sex and RH. Male and female are only adjusted for age and RH. CI = confidence interval.

## Data Availability

National Health Insurance Research Database, Taiwan. Available online: http://nhird.nhri.org.tw/en/index.htm (accessed on 5 May 2022).

## References

[1] McInnes I.B., Schett G. (2011). The pathogenesis of rheumatoid arthritis. N. Engl. J. Med..

[2] Cross M., Smith E., Hoy D., Carmona L., Wolfe F., Vos T., Williams B., Gabriel S., Lassere M., Johns N. (2014). The global burden of rheumatoid arthritis: Estimates from the global burden of disease 2010 study. Ann. Rheum. Dis..

[3] Almutairi K., Nossent J., Preen D., Keen H., Inderjeeth C. (2021). The global prevalence of rheumatoid arthritis: A meta-analysis based on a systematic review. Rheumatol. Int..

[4] Myasoedova E., Crowson C.S., Kremers H.M., Therneau T.M., Gabriel S.E. (2010). Is the incidence of rheumatoid arthritis rising?: Results from Olmsted County, Minnesota, 1955–2007. Arthritis Rheumatol..

[5] Hunter T.M., Boytsov N.N., Zhang X., Schroeder K., Michaud K., Araujo A.B. (2017). Prevalence of rheumatoid arthritis in the United States adult population in healthcare claims databases, 2004–2014. Rheumatol. Int..

[6] Lai C.H., Lai M.S., Lai K.L., Chen H.H., Chiu Y.M. (2012). Nationwide population-based epidemiologic study of rheumatoid arthritis in Taiwan. Clin. Exp. Rheumatol..

[7] Arend W.P., Firestein G.S. (2012). Pre-rheumatoid arthritis: Predisposition and transition to clinical synovitis. Nat. Rev. Rheumatol..

[8] Linn-Rasker S.P., van der Helm-van Mil A.H., van Gaalen F.A., Kloppenburg M., de Vries R.R., le Cessie S., Breedveld F.C., Toes R.E., Huizinga T.W. (2006). Smoking is a risk factor for anti-CCP antibodies only in rheumatoid arthritis patients who carry HLA-DRB1 shared epitope alleles. Ann. Rheum. Dis..

[9] Mikuls T.R., Payne J.B., Yu F., Thiele G.M., Reynolds R.J., Cannon G.W., Markt J., McGowan D., Kerr G.S., Redman R.S. (2014). Periodontitis and Porphyromonas gingivalis in patients with rheumatoid arthritis. Arthritis Rheumatol..

[10] Demoruelle M.K., Weisman M.H., Simonian P.L., Lynch D.A., Sachs P.B., Pedraza I.F., Harrington A.R., Kolfenbach J.R., Striebich C.C., Pham Q.N. (2012). Brief report: Airways abnormalities and rheumatoid arthritis-related autoantibodies in subjects without arthritis: Early injury or initiating site of autoimmunity?. Arthritis Rheumatol..

[11] Hutchinson D., Moots R. (2001). Cigarette smoking and severity of rheumatoid arthritis. Rheumatology.

[12] Pedersen M., Jacobsen S., Klarlund M., Pedersen B.V., Wiik A., Wohlfahrt J., Frisch M. (2006). Environmental risk factors differ between rheumatoid arthritis with and without auto-antibodies against cyclic citrullinated peptides. Arthritis Res. Ther..

[13] Makrygiannakis D., Hermansson M., Ulfgren A.K., Nicholas A.P., Zendman A.J., Eklund A., Grunewald J., Skold C.M., Klareskog L., Catrina A.I. (2008). Smoking increases peptidylarginine deiminase 2 enzyme expression in human lungs and increases citrullination in BAL cells. Ann. Rheumatol. Dis..

[14] Katsouyanni K. (2003). Ambient air pollution and health. Br. Med. Bull..

[15] Zhao C.N., Xu Z., Wu G.C., Mao Y.M., Liu L.N., Qian W., Dan Y.L., Tao S.S., Zhang Q., Sam N.B. (2019). Emerging role of air pollution in autoimmune diseases. Autoimmun. Rev..

[16] Sigaux J., Biton J., André E., Semerano L., Boissier M.C. (2019). Air pollution as a determinant of rheumatoid arthritis. Jt. Bone Spine.

[17] Perricone C., Versini M., Ben-Ami D., Gertel S., Watad A., Segel M.J., Ceccarelli F., Conti F., Cantarini L., Bogdanos D.P. (2016). Smoke and autoimmunity: The fire behind the disease. Autoimmun. Rev..

[18] Hart J.E., Laden F., Puett R.C., Costenbader K.H., Karlson E.W. (2009). Exposure to traffic pollution and increased risk of rheumatoid arthritis. Environ. Health Perspect..

[19] De Roos A.J., Koehoorn M., Tamburic L., Davies H.W., Brauer M. (2014). Proximity to traffic, ambient air pollution, and community noise in relation to incident rheumatoid arthritis. Environ. Health Perspect..

[20] Chen C.-H., Wu C.-D., Chiang H.-C., Chu D., Lee K.-Y., Lin W.-Y., Yeh J.-I., Tsai K.-W., Guo Y.-L.L. (2019). The effects of fine and coarse particulate matter on lung function among the elderly. Sci. Rep..

[21] Genc S., Zadeoglulari Z., Fuss S.H., Genc K. (2012). The adverse effects of air pollution on the nervous system. J. Toxicol..

[22] Puett R.C., Hart J.E., Suh H., Mittleman M., Laden F. (2011). Particulate matter exposures, mortality, and cardiovascular disease in the health professionals follow-up study. Environ. Health Perspect..

[23] Guo X., Lin Y., Lin Y., Zhong Y., Yu H., Huang Y., Yang J., Cai Y., Liu F., Li Y. (2022). PM2.5 induces pulmonary microvascular injury in COPD via METTL16-mediated m6A modification. Environ. Pollut..

[24] Hart J.E., Källberg H., Laden F., Bellander T., Costenbader K.H., Holmqvist M., Klareskog L., Alfredsson L., Karlson E.W. (2013). Ambient air pollution exposures and risk of rheumatoid arthritis: Results from the Swedish EIRA case-control study. Ann. Rheum. Dis..

[25] Hart J.E., Källberg H., Laden F., Costenbader K.H., Yanosky J.D., Klareskog L., Alfredsson L., Karlson E.W. (2013). Ambient air pollution exposures and risk of rheumatoid arthritis. Arthritis Care Res..

[26] Chang K., Yang S.M., Kim S.H., Han K.H., Park S.J., Shin J.I. (2014). Smoking and rheumatoid arthritis. Int. J. Mol. Sci..

[27] Heliövaara M., Aho K., Aromaa A., Knekt P., Reunanen A. (1993). Smoking and risk of rheumatoid arthritis. J. Rheumatol..

[28] Barr J., Sharma C.S., Sarkar S., Wise K., Dong L., Periyakaruppan A., Ramesh G.T. (2007). Nicotine induces oxidative stress and activates nuclear transcription factor kappa B in rat mesencephalic cells. Mol. Cell Biochem..

[29] Bracke K., Cataldo D., Maes T., Gueders M., Noël A., Foidart J.M., Brusselle G., Pauwels R.A. (2005). Matrix metalloproteinase-12 and cathepsin D expression in pulmonary macrophages and dendritic cells of cigarette smoke-exposed mice. Int. Arch. Allergy Immunol..

[30] Chang K.H., Hsu C.C., Muo C.H., Hsu C.Y., Liu H.C., Kao C.H., Chen C.Y., Chang M.Y., Hsu Y.C. (2016). Air pollution exposure increases the risk of rheumatoid arthritis: A longitudinal and nationwide study. Environ. Int..

[31] Bernatsky S., Smargiassi A., Joseph L., Awadalla P., Colmegna I., Hudson M., Fritzler M.J. (2017). Industrial air emissions, and proximity to major industrial emitters, are associated with anti-citrullinated protein antibodies. Environ. Res..

[32] Zhao N., Smargiassi A., Hatzopoulou M., Colmegna I., Hudson M., Fritzler M.J., Awadalla P., Bernatsky S. (2020). Long-term exposure to a mixture of industrial SO_2_, NO_2_, and PM2.5 and anti-citrullinated protein antibody positivity. Environ. Health.

[33] Park J.S., Choi S., Kim K., Chang J., Kim S.M., Kim S.R., Lee G., Son J.S., Kim K.H., Lee E.Y. (2021). Association of particulate matter with autoimmune rheumatic diseases among adults in South Korea. Rheumatology.

[34] Park M., Joo H.S., Lee K., Jang M., Kim S.D., Kim I., Borlaza L.J.S., Lim H., Shin H., Chung K.H. (2018). Differential toxicities of fine particulate matters from various sources. Sci. Rep..

[35] Essouma M., Noubiap J.J. (2015). Is air pollution a risk factor for rheumatoid arthritis?. J. Inflamm..

[36] van der Woude D., Catrina A.I. (2015). HLA and anti-citrullinated protein antibodies: Building blocks in RA. Best Pract. Res. Clin. Rheumatol..

[37] Wu Q., Xu Z., Dan Y.L., Cheng J., Zhao C.N., Mao Y.M., Xiang K., Hu Y.Q., He Y.S., Pan H.F. (2021). Association between traffic-related air pollution and hospital readmissions for rheumatoid arthritis in Hefei, China: A time-series study. Environ. Pollut..

[38] Di D., Zhang L., Wu X., Leng R. (2020). Long-term exposure to outdoor air pollution and the risk of development of rheumatoid arthritis: A systematic review and meta-analysis. Semin. Arthritis Rheumatol..

[39] Adami G., Viapiana O., Rossini M., Orsolini G., Bertoldo E., Giollo A., Gatti D., Fassio A. (2021). Association between environmental air pollution and rheumatoid arthritis flares. Rheumatology.

[40] Hwa-Lung Y., Chih-Hsin W. (2010). Retrospective prediction of intraurban spatiotemporal distribution of PM2.5 in Taipei. Atmos. Environ..

